# Efficacy of capacitive resistive monopolar radiofrequency in the physiotherapeutic treatment of chronic pelvic pain syndrome: study protocol for a randomized controlled trial

**DOI:** 10.1186/s13063-021-05321-6

**Published:** 2021-05-20

**Authors:** A. Carralero-Martnez, M. A. Muoz Prez, R. Pan-Alemany, L. Blanco-Ratto, S. Kauffmann, I. Ramrez-Garca

**Affiliations:** 1 Rehabilitacin Abdomino-Pelviana (RAPbarcelona SL), Barcelona, Spain; 2 Servicio de Ginecologa, Instituto Clnic de Ginecologa, Obstetricia y Neonatologa, Hospital Clnic de Barcelona, Barcelona, Spain; 3 Institut Universitari dInvestigaci en Atenci Primria Jordi Gol (IDIAP-Jordi Gol), Barcelona, Spain; 4 Institut Catal de la Salut (ICS), Barcelona, Spain; 5 Departament de Pediatria, Obstetricia i Ginecologia i Medicina Preventiva, Universitat Autnoma de Barcelona (UAB), Bellaterra, Spain; 6 Fundaci Universitria del Bages (FUB), Barcelona, Spain; 7 Servicio de Fisioterapia, Womens Salud y Bienestar de la Mujer SL, Barcelona, Spain; 8 Servicio de Fisioterapia, Instituto Mdico Tecnolgico SL, Barcelona, Spain; 9 Blanquerna School of Health Science-Universitat Ramon Llull, Barcelona, Spain; 10 Universidad Internacional de Catalunya (UIC), Barcelona, Spain

**Keywords:** Chronic pelvic pain syndrome, Musculoskeletal pain, Physical therapy, Therapeutic interventions, Capacitive resistive monopolar radiofrequency, Randomized controlled trial, Gynecology, Urology

## Abstract

**Background:**

Chronic pelvic pain syndrome (CPPS) is a multifactorial disorder that affects 5.7% to 26.6% of women and 2.2% to 9.7% of men, characterized by hypersensitivity of the central and peripheral nervous system affecting bladder and genital function. People with CPPS have much higher rates of psychological disorders (anxiety, depression, and catastrophizing) that increase the severity of chronic pain and worsen quality of life. Myofascial therapy, manual therapy, and treatment of trigger points are proven therapeutic options for this syndrome. This study aims to evaluate the efficacy of capacitive resistive monopolar radiofrequency (CRMRF) at 448kHz as an adjunct treatment to other physiotherapeutic techniques for reducing pain and improving the quality of life of patients with CPPS.

**Methods:**

This triple-blind (1:1) randomized controlled trial will include 80 women and men with CPPS. Participants will be randomized into a CRMRF activated group or a CRMRF deactivated group and receive physiotherapeutic techniques and pain education. The groups will undergo treatment for 10 consecutive weeks. At the beginning of the trial there will be an evaluation of pain intensity (using VAS), quality of life (using the SF-12), kinesiophobia (using the TSK-11), and catastrophism (using the PCS), as well as at the sixth and tenth sessions.

**Discussion:**

The results of this study will show that CRMRF benefits the treatment of patients with CPPS, together with physiotherapeutic techniques and pain education. These results could offer an alternative conservative treatment option for these patients.

**Trial registration:**

ClinicalTrials.gov NCT03797911. Registered on 8 January 2019.

**Supplementary Information:**

The online version contains supplementary material available at 10.1186/s13063-021-05321-6.

## Background

Chronic pelvic pain syndrome (CPPS) is defined as pain of non-oncological cause, intermittent or constant, in the lower part of the abdomen or pelvis, in both men and women, lasting at least 6months, and with negative consequences that can be cognitive, behavioral, sexual and emotional [1, 2]. It is a multifactorial disorder serious enough to cause urinary and genital functional disability and with high prevalence rates (5.7% to 26.6% of women and 2.2% to 9.7% of men) [3,8].

People with CPPS have much higher rates of psychological distress. The prevalence of anxiety ranges between 39 and 73%, compared to 12% of the general population, while depression is seen in 26 to 52%, compared to between 5 and 10% of the general population [9,13]. These conditions, along with catastrophizing, are associated with increased severity of chronic pain and reduced quality of life [4, 14,18].

In addition, people with CPPS tend to have central and peripheral nervous system hypersensitivity, with dysfunctional pain modulation that tends to aggravate pain [19,24].

In physical therapy consultations, there are a variety of therapeutic options with sufficient evidence to guide physical therapy for patients with CPPS [25]. The most widely used is myofascial therapy, although these patients should be treated using a multidisciplinary approach including other therapies such as psychology, medication, or surgery when other treatments have failed [26, 27]. One such option in clinical practice is capacitive resistive monopolar radiofrequency (CRMRF) at 448kHz. This non-invasive strategy increases the temperature of deep organs or tissues using radiofrequency electrical currents to reduce pain and inflammation and increase tissue repair [28,35]. Few recent studies evaluate its clinical efficacy despite being common practice for the last 20years [36]. Current studies report promising results in terms of pain reduction and improved function in musculoskeletal pathologies (such as low back pain) [37] and tendinopathies (such as plantar fasciitis) [38,40].

Despite its demonstrated efficacy in other musculoskeletal pathologies, there is currently insufficient scientific evidence regarding its role in the management of CPPS.

## Methods

### Aim

The superiority study hypothesizes that the application of CRMRF associated with physiotherapy techniques and health education provides benefits in reducing pain when compared to physiotherapy and health education techniques alone in patients with CPPS.

The specific aims are to evaluate the efficacy of the CRMRF according to the intensity of pain, quality of life, kinesiophobia, and catastrophism of patients participating in the study. In addition, sociodemographic and clinical data, adherence to treatment, and possible adverse events during treatment will be recorded for both groups.

### Design

This manuscript describes a research protocol for a triple-blind, randomized controlled clinical trial. Participants will be equally (1:1) and randomly allocated into either an activated capacitive resistive monopolar radiofrequency group (intervention group, IG) or a deactivated capacitive resistive monopolar radiofrequency group (control group, CG). Both groups will receive pain education and physiotherapeutic techniques (myofascial therapy, trigger point therapy, and/or manual therapy). Participants, the investigators performing the intervention, and the statistical analyses will be blinded.

An analysis of the results will be carried out at 6 and 10weeks of treatment.

### Location

This trial will take place at RAPbarcelona, a pelvic floor specialized physiotherapy center in Barcelona.

### Participants

Patients receiving their first consultation at the center, or those referred by other health professionals familiar with the protocol of this study, will be invited to participate.

To be eligible, participants must meet the following inclusion criteria are of legal age, have suffered from CPPS for 6months or more (etiologies will include myofascial syndrome, endometriosis, adenomyosis, inflammatory prostatitis, bladder pain syndrome, levator ani syndrome, pudendal nerve syndrome, or nonspecific CPPS), and agree to participate in the study granting signed informed consent. Exclusion criteria include undergoing other conservative treatments during the study (manual therapy, physical therapy, osteopathy, chiropractic, massage), having undergone treatment with chemotherapy or radiotherapy in the pelvic area, having recently undergone an oncological process, being pregnant, having undergone surgery in the pelvic area in the last 3months, presenting fibromyalgia or chronic fatigue, suffering serious psyche disorders, presenting hypersensitivity in the skin that may be in contact with the treatment, or suffering from neuromuscular diseases.

### Procedure

Patients who agree to participate in the study will receive CRMRF therapy for 30min once a week. The first session, lasting between 45 and 60min, will serve to inform the patient, obtain signed informed consent, resolve any doubts about the study or questionnaires, and explain the theory of pain and health education.

At baseline, participants will undergo an initial assessment where data on age, medical history, surgical history, and clinical data will be collected. According to criteria used in previous similar studies [41, 42] palpation of the abdominal, lumbosacral, and perineal region will be performed, followed by internal palpation using the index finger at the vaginal and/or anal level to palpate the pelvic floor muscles, connective tissue, and internal organs, and to localize pain. During the first visit, the visual analog scale (VAS) will be used to measure the intensity of pain, the health questionnaire Short Form 12 (SF-12) to assess the quality of life, the Tampa Scale for Kinesiophobia (TSK-11) to assess kinesiophobia, and the Pain Catastrophizing Scale (PCS) to assess catastrophism. The same tests will be re-evaluated after 6 and 10 sessions. Also, at each session, treatment adherence and possible adverse events of the therapy will be identified and recorded in a database designed for the project.

The most common adverse reaction to the application of CRMRF, particularly at the beginning of treatment, is an increase in pain in the area lasting approximately 2 to 3days, which can be controlled with the local application of heat and the use of oral analgesics. In rare cases, due to the local application of heat, dermal irritation could appear. In this case, topical treatments may be applied. If the dermal irritation persists in the next session (after 1 week), it will be noted on the patients record sheet and the intervention will cease until it is completely resolved.

A total of 10 treatment physiotherapy sessions will be held weekly (Table 1).

**Table 1:** Treatment sessions

	Intervention group (IG)	Control group (CG)
Session 1	Review of the information sheet and signing of informed consent. Collection and recording of baseline data (age, sociodemographic data, clinical data, and medical and surgical history). Self-completion of tests (VAS, SF-12, TSK-11, and PCS).	
	Application of physiotherapeutic techniques with activated CRMRF. Explanation of the theory of pain and health education.	Application of physiotherapeutic techniques with deactivated CRMRF. Explanation of the theory of pain and health education.
Session 25	Session protocol: Registration of possible discomfort or adverse events perceived by the patient. Application of physiotherapeutic techniques with activated CRMRF. Pain education clarifications	Session protocol: Registration of possible discomfort or adverse events perceived by the patient. Application of physiotherapeutic techniques with deactivated CRMRF. Pain education clarifications
Session 6	Collection of VAS, SF-12, TSK-11, and PCS tests. Session protocol (as described in session 25)	
Session 79	Session protocol (as described in session 25)	
Session 10	Session protocol (as described in session 25) Assessment of VAS, SF-12, TSK-11, PCS tests, evolution of the pathology, and referral (if required).	

Both groups will follow the same protocol, which will consist of applying the CRMRF (INDIBA Activ CT8, INDIBA S.A, Sant Quirze del Valls, Barcelona, Spain) at 2% to induce an electrical and athermic effect, along with pain education and physiotherapeutic techniques [43, 44] administered at the same time as the CRMRF according to the location of the pain (Table 2). Participants will be placed comfortably in a supine or prone position (depending on the area to be treated) with a pillow under their heads, undressed from the waist down. The plate will be placed on the abdomen or lower back depending on the patients position, and the 32mm resistive electrode will be used to apply the CRMRF to the affected area.

**Table 2:** Physiotherapeutic techniques and position of the patient during treatment sessions, depending on the location of the pain

	Anterior location (abdomen, pubis, groin, perineum, vagina, penis, testicles)	Posterior location (lumbar, sacrum, coccyx, buttocks, anus, rectum)
Position:	Patient in supine position. CRMRF plate on lower back	Patient in the prone position. CRMRF plate on abdomen
Techniques:	Abdominal area: - Lift techniques of the peritoneum - Liberation of the urachus Groin area: - Stretching the inguinal ligament - Myotensive techniques of the internal obturator Vulvar, perineal and vaginal area: - Relaxation of the superficial fascia of the perineum - Stretching the prevesical ligament - Uterine release techniques - Stretching of the round ligament - Stretching of the wide ligament - Relaxation of the sacrorectogenitopubian laminae - Release of the pudendal nerve in Alcocks canal Penis and testicular area: - Relaxation of the superficial fascia of the perineum - Relaxation of the deep fascia of the perineum - Testicular drainage	Lumbosacral area: - Relaxation of the quadratus lumbar - Relaxation of the paravertebral muscles Gluteal area: - Decompression of the pudendal nerve in the greater sciatic foramen - Stretching of the sacrociatic ligament - Stretching of the sacrotuberous ligament - Release of the pudendal nerve in the ischiorectal fossa - Myotensive techniques of the pyramidal - Myotensive techniques of the external obturator Anorectal area: - Sacral plexus release techniques - Relaxation of the sacrorectogenitopubian laminae - Stretching the Denonvilliers fascia - Prostate release techniques
	If there is scarring, manual scar work is performed, and the 35mm resistive electrode is applied over it.	

Participants in the IG will receive treatment with the activated CRMRF (emitting electrical signal) while participants in the CG will receive the same treatment with the deactivated CRMRF (not emitting electrical signal).

This CRMRF equipment, specifically modified for research purposes, is designed to perform both the conventional treatment (IG) and a placebo treatment (CG) without any visible difference to either the therapist or the participant. The equipment, fully adapted for our research, produces automatic randomization for each participant according to the order of the study assignment.

Pain education will consist of basic theory about gate control [45], concepts of pain and central sensitization, and a basic explanation of the neurotransmitters that influence the increase or decrease in pain [46,48].

The physiotherapeutic techniques that will be performed in each session will be the same in all individualized treatment sessions for each patient and assigned according to the location of the pain (Table 2). These techniques will be those recommended by the literature for the treatment of CPPS and will consist of myofascial induction techniques, trigger point therapy, and manual therapy with the aim of improving the elasticity of the musculature and fascial tissue and improving blood flow. They will be performed with smooth, slow, and increasingly direct movements, beginning distally and becoming more localized [49]. This information will be emphasized to the patient to improve treatment and follow-up, as well as the fact that this ordinarily costly treatment will have no cost for study participants.

As this is a study with multiple tests and interventions, several physical therapists will be needed to carry it out. For this reason, and to avoid errors due to lack of standardization, there will be training in the application of the therapy and data collection for all physiotherapists who participate in the study.

### Outcome measures

Participants will complete three study assessments: a baseline assessment and at 6 and 10weeks after the first session.

Primary outcome:

- The intensity of pain: According to the VAS score, which will be evaluated in the first, sixth, and tenth sessions of the study. This quantitative and subjective variable consists of marking the degree of pain intensity in a straight horizontal line of a fixed length of 10cm. The ends are defined as the extreme limits of the parameter to be measured from left (worst) to right (best) [50, 51].

Secondary outcomes:

- The quality of life related to health: Measured with the SF-12 Quality of Life health questionnaire. Specifically, the Spanish adaptation of the SF-12 Health Survey [52, 53] done by Alonso et al. [54, 55] will be used. The SF-12 is a reduced version of the SF-36 Health Questionnaire designed for cases in which a shorter questionnaire is required. While the SF-12 can be answered in an average of 2min, the SF-36 takes between 5 and 10min to complete. It consists of 12 items from the 8 dimensions of the SF-36 (physical function, social function, physical role, emotional role, mental health, vitality, body pain, and general health status). A higher score means better quality of life.
- The kinesiophobia: Fear of movement will be measured by the Tampa Scale for Kinesiophobia (TSK-11). This questionnaire, created by Miller et al. [56], quantifies the intensity of kinesiophobia suffered by the patient. The reduced version and adaptation to Spanish by Gmez-Prez et al. [57] will be used. It consists of 11 statements that the patient must answer using a Likert-type scale from 1 (totally disagree) to 4 (totally agree). A higher score means a greater degree of kinesiophobia.
- The catastrophizing: Measured with the Pain Catastrophizing Scale (PCS) [58]. This 13-item questionnaire assesses the patient's catastrophic thoughts using a five-point Likert scale. It consists of three subscales (rumination, magnification, and hopelessness). A higher score means a greater degree of catastrophizing. The Spanish adaptation validated by Garca Campayo et al. [59] will be used.
- Sociodemographic variables, pathological history, and clinical history: Assessed in the first treatment session and collected through a standardized clinical history.
- Adverse events: Recorded in each of the treatment sessions through patient references to his or her status and evolution.
- Adherence to treatment: Assessed in each of the treatment sessions and collected through a compliance form designed for the project.

### Schedule Table 3

**Table 3:** The different schedule phases are shown in italics

	Recruitment	Session 1	Sessions 25	Session 6	Sessions 79	Session 10
**Recruitment:**						
Selection screening	X					
Informed consent	X					
Allocation	X					
**Interventions:**						
Intervention CG (deactivated CRMRF		X	X	X	X	X
Intervention IG (activated CRMRF)		X	X	X	X	X
**Evaluations:**						
Demographic variables		X				
Clinical variables		X				
VAS		X		X		X
SF-12		X		X		X
TSK-11		X		X		X
PCS		X		X		X
Compliance form		X	X	X	X	X
Recording of adverse events		X	X	X	X	X

### Sample size

To estimate the sample size, version 7.12 (April 2012) of the sample size calculator of the GRANMO program was used. This version can be obtained at the following link: https://www.imim.es/ofertadeserveis/software-public/granmo/.

For this estimate, alpha values of 5% and beta values of 20% (power of 80%) were taken into account. Based on data published in the literature [60] and applying a common standard deviation of 3 and a difference equal to or greater than 2 in the VAS, 40 patients are needed for each leg of the study, assuming a maximum percentage of follow-up losses/dropouts of 10%.

### Selection of the sample

The selection of the sample will be done by sampling consecutive cases from the RAPbarcelona clinic in Barcelona. Health professionals from other institutions will be contacted to increase referrals to the center and an advertising campaign will be carried out on social networks. In physiotherapist appointments, patients with CPPS will be referred to the principal investigator. The protocol will be clearly explained to each of the interested patients who meet the selection criteria, and patients will be asked to sign an informed consent if they choose to participate. Patients will then be allocated to one of the two study groups.

### Random allocation of groups

Once participants are included in the study, they will be identified by the number from their computerized medical record and will be ordered sequentially and consecutively from 1 to 80 according to the order of recruitment. To assign interventions, CRMRF team engineers will enter the randomized sequence corresponding to each number from 1 to 80 into the study software to designate the CG and IG participants. In order to keep the assigned study group hidden from the patient, the physiotherapist, and the main researcher, the following four indications will be taken into account: (1) No parameter will appear on the screen visible to the CRMRF team that indicates whether the equipment emits an electrical signal. (2) The current intensity parameter will be 2% for all participants to prevent the intervention group patients from perceiving any thermal effect. (3) The physiotherapists will apply the CRMRF by manipulating the equipment with the handle and never with the electrode to avoid any sensation. They will be trained before starting the study. (4) The sequence of randomization and allocation will be kept hidden at all times from all patients, professionals, and the main researcher until the statistical analysis once the intervention is completed.

### Collection, management, and data analysis

Data will be collected in a specific database coded for this study, which will only be available to the main researcher. The database and statistical analysis will be performed with IBM SPSS Statistics 24.0 software.

First, a descriptive analysis of the characteristics of the patients included in both study groups, as well as the outcome variables will be carried out. To do this, absolute and relative frequencies (percentages) will be estimated for qualitative variables, and mean or median and standard deviation or range, respectively, depending on the normality of the distribution, for quantitative variables. Then, the comparative analysis of the two treatment groups will be carried out using the Chi-square test for qualitative variables and the Students *t* test for quantitative variables. Additionally, different associations between diverse variables will be analyzed. To check for the efficacy of the study treatments, intention to treat (ITT) and by protocol (PP) analysis will be performed.

The comparison of results will be done by promptly estimating the differences of the mean values of the outcome variables with their corresponding 95% confidence intervals (95% CI) and standard deviation (SD). Additionally, the adjusted differences will be calculated following the indications of the CONSORT document [61].

All data will be systematically included in the statistical database and an analysis of the results will be carried out individually for each participant both in the middle of the treatment (fifth session) and at the end (tenth session) to evaluate the evolution. The trial will cease if significant worsening, absolute improvement (VAS = 0), or unknown serious adverse events occur. A full preliminary results analysis will be performed when one-third of the sample size has been reached and the final analysis will be performed once the last participant has completed the intervention.

In all cases, the level of statistical significance established will be the usual (5%); therefore, statistically significant differences will be considered when p values are less than 0.05.

## Discussion

The results of this study will make it possible to prove that CRMRF benefits the treatment of patients with CPPS together with physiotherapeutic techniques and pain education. These results could offer an alternative conservative treatment option for these patients.

## Trial status

This is the first version of the protocol (January 28, 2021). Recruitment began in April 2019 and the intervention was put on hold due to the coronavirus crisis. For this reason, completion is expected by next April.

## Supplementary Information

**Additional file 1.**

## Data Availability

Not applicable.

## Abbreviations

CG: Control group
CPPS: Chronic pelvic pain syndrome
CRMRF: Capacitive resistive monopolar radiofrequency
IG: Intervention group
PCS: Pain Catastrophizing Scale
SF-12: Health Questionnaire Short Form 12
TSK-11: Tampa Scale for Kinesiophobia
VAS: Visual analog scale
