# Supplementary material for: Efficacy of capacitive resistive monopolar radiofrequency in the physiotherapeutic treatment of chronic pelvic pain syndrome: study protocol for a randomized controlled trial
Source: Trials. 2021 May 20;22:356. doi: 10.1186/s13063-021-05321-6 (PMC8136758; doi:10.1186/s13063-021-05321-6)
Supplement: Supplementary file 1 — Additional file 1. [file 13063_2021_5321_MOESM1_ESM.docx]

# Appendices

**Informed Consent**

Title of study: Efficacy of capacitive resistive monopolar radiofrequency in the physiotherapeutic treatment of chronic pelvic pain syndrome: a randomized controlled trial.

I, ___________________________________________________ (name and surname),

with ID card number ___________________________, I declare that:

I have read the information sheet that has been given to me.

I have been able to ask questions about the study.

I have discussed it with Ms. Andrea Carralero Martinez (Principal Investigator).

I understand that my participation is voluntary at all times.

I understand that I can withdraw from the study:

- Whenever I wish
- Without having to give any explanation
- Without it affecting my medical care

In accordance with the provisions of the L.O. 15/1999, of December 13 on data protection of personal character, I declare to have been informed of the purpose of collecting my data and the recipients of the information.

Thus, I freely agree to participate in this study.

Barcelona, on ______________20___

Participant Signature: Principal Investigator Signature:

**Patient Information:** Randomized Study of Non-Invasive Procedure

Research project titled: Efficacy of capacitive resistive monopolar radiofrequency in the physiotherapeutic treatment of chronic pelvic pain syndrome: randomized controlled trial.

Principal Investigator: Sra. Andrea Carralero Martinez

Centro de Rehabilitación Abdomino-Pelviana RAPbarcelona SL. Av. Diagonal 363, 3º 2ª (08037 BARCELONA)

Sponsor: RAPbarcelona S.L.

Name of patient: ________________________________________________________

**Goals:**

We request your participation in this research project whose main objective is to evaluate the efficacy of Resistive Capacitive Monopolar Radiofrequency (RCMRF) in patients with Chronic Pelvic Pain Syndrome (CPPS). This technique is practiced in physiotherapy consultations for the pelvic floor and is currently used for the treatment of this pathology and others, together with manual techniques that constitute standard pelvic floor physiotherapeutic treatment.

**Benefits:**

You may not benefit directly from participating in this study. However, the evaluation of these strategies related to chronic pelvic pain could benefit future patients who suffer from CPPS and contribute to a better understanding and treatment of this pathology.

**Possible risks:**

Participants are patients with CPPS lasting for more than 6 months. The efficacy of RCMRF for this pathology will be evaluated. The therapy is carried out for 45 minutes using a radiofrequency equipment. After applying the treatment, it is normal for the patient to perceive a thermal sensation in the treated area. You may also perceive transient temporary discomfort in the area. On rare occasions, the application of the cream may cause mild skin irritation.

**Precautions:**

If you suffer from any of the contraindications or precautionary reasons listed below, please notify the research team members who are caring for you:

• If you are hypotensive you may experience a drop in blood pressure. In this case, the treatment will cease until normal blood pressure levels are restored.

• If you are undergoing oral anticoagulant therapy you should consult your doctor before starting the treatment.

• If you have a known allergy to nickel or chromium, take special care. Stop treatment if an allergic reaction appears in the areas of contact with the return plate, the return cylinder, or resistive electrodes.

• If you have an internal infection and encapsulation (abscesses) you may be at risk of spreading the infection. Do not begin treatment without first consulting your doctor.

• Special caution must be exercised with elderly patients who may present cutaneous alterations to avoid dermal injuries or burns.

**Study procedures:**

This study aims to evaluate the effectiveness of RCMRF as a complement to conventional physiotherapy treatment using and comparing two different procedures.

The patient’s assigned treatment will be random. Your doctor will not be involved in this process. You will have a 50% probability of receiving each of the procedures carried out in this study.

In both cases, the therapy consists of receiving conventional standardized physiotherapy treatment for patients with CPPS. This includes myofascial techniques, manual therapy for trigger points, and health education for pain management for 10 consecutive weeks with the objective of modifying pain intensity and improving the quality of life. Additionally, and while performing the previously mentioned techniques, the RCMRF will be applied with capacitive resistance activated or deactivated, depending on the assigned group. These treatments are performed by a physiotherapist with no effort required by participants. Patients will be asked simply to attend the sessions and relax during the interventions. During all sessions, your attendance, information, and any noteworthy observations will be recorded. In addition to the 45-minute treatment sessions, the following data will be collected via questionnaires as described below.

Inclusion visit: During the inclusion visit, your personal, demographic data will be collected. Medical history and pain history: Age, sex, socioeconomic status, medical history, pharmacological treatment, health habits (exercise, tobacco, alcohol, and drug use), obstetric history, location and duration of pain, any gastrointestinal, urinary or pelvic symptoms, and history of sexual abuse.

Once this procedure has been carried out, and prior to beginning treatment, the intensity of pain will be measured using the Visual Analogue Scale (VAS). You will also be asked to fill out the SF-12 questionnaire (Short Form 12 - Quality of life), the TSK-11 questionnaire (Tampa Scale of Kinesiophobia), and the ECD questionnaire (Catastrophism Scale) at home, which will be explained in detail.

Visit 1: Treatment will begin, questionnaires will be collected, and patients will be asked to assess their pain using the VAS (Visual Analogue Scale).

Visits 2-4: These will be weekly 30-minute visits during which physiotherapy techniques appropriate to the location of pain will be applied either with or without RCMRF These techniques are performed specifically according to the location of your pain and are applied externally in the pelvic area (abdomen, pubis, perineum, buttocks, lumbar) and intracavitary (internally in vagina and anus).

Visit 5: The usual treatment will be carried out and questionnaires returned.

Visit 6: The questionnaires completed at home will be collected, the VAS will be requested, and the usual treatment will be carried out.

Visits 7-9: The treatment will be carried out and the adherence/incident record will be completed at the end of the session.

Visit 10: This will be the final treatment session and the VAS will be requested for the last time. The SF-12, TSK-11, and ECD questionnaires will be returned, and the current state of health will be discussed in order to proceed with the prescription of the therapy that best suits the new needs of the patient/study participant.

**Personal data protection:**

In accordance with Regulation (EU) 2016/679 of the European Parliament and of the council of April 27, 2016 protection of personal data and the free circulation of these data, and the Royal Decree-law 5/2018, of July 27, of urgent measures for the adaptation of Spanish law to the regulations of the European Union in terms of data protection, the personal data obtained will be that necessary to meet the purposes of the study. Your name will not appear in any of the study reports and your identity will not be revealed to anyone except to fulfill the purposes of the study and in the case of a medical emergency or legal requirement. Any personal information that may be identifiable will be kept digitally under secure conditions by the principal investigator of the study, Ms. Andrea Carralero Martinez. Access to such information will be restricted to RAPbarcelona staff designated for this purpose or to other authorized staff who will be obliged to maintain the confidentiality of the information.

In accordance with current law, you have the right to access your personal data. Also, and when justified, you have the right to rectification and cancellation, which you should request from the doctor caring for you during the study.

In accordance with current legislation, you have the right to be informed of relevant health data obtained in the course of the study. This information will be communicated to you if you wish; in the case that you prefer not to be informed, your decision will be respected.

If you need more information about this study you can contact the responsible researcher, Andrea Carralero Martinez of the Physiotherapy Service / Tel. 934582341

Your participation in the study is completely voluntary and if you choose not to participate you will still receive all required care and your relationship with the medical team caring for you will not be affected.
